# Dyslipidemia awareness, treatment, control and influence factors among adults in the Jilin province in China: a cross-sectional study

**DOI:** 10.1186/1476-511X-13-122

**Published:** 2014-08-03

**Authors:** Huan He, Ya-qin Yu, Yong Li, Chang-gui Kou, Bo Li, Yu-chun Tao, Qing Zhen, Chang Wang, Joseph Sam Kanu, Xu-feng Huang, Mei Han, Ya-wen Liu

**Affiliations:** 1 Department of Epidemiology and Biostatistics, School of Public Health, Jilin University, Changchun 130021, China; 2 Illawarra Health and Medical Research Institute, University of Wollongong, Wollongong NSW2522, Australia

**Keywords:** Dyslipidemia, Awareness, treatment and control, Influence factors

## Abstract

**Background:**

In China, even though the prevalence of dyslipidemia among adults increased yearly and dyslipidemia being an important risk factor for cardiovascular diseases among the Chinese population, however, the awareness, treatment and control of dyslipidemia are at low levels, and only limited studies on the influence factors associated with the awareness, treatment and control dyslipidemia in China have been carried out.

**Methods:**

The analysis was based on a representative sample of 7138 adult subjects aged 18 ~ 79 years recruited from a cross-sectional study of chronic disease and risk factors among adults in the Jilin province in 2012. *Chi-square* test was used to compare the rates of dyslipidemia awareness, treatment and control between different characteristics of participants. Multiple logistic regression analyses were performed separately for each group to explore the associations between participants’ characteristics and dyslipidemia awareness, treatment and control.

**Results:**

Among participants with dyslipidemia, 11.6% were aware of the diagnosis, 8.4% were receiving treatment, and 34.8% had dyslipidemia controlled. Increase in age and BMI ≥ 24 kg/m^2^ were by far the strongest risk factors associated with better awareness and treatment of dyslipidemia. Retirees were more likely to be aware of their dyslipidemia condition (OR = 1.255; 95% CI: 1.046, 1.506) and to be receiving treatment (OR = 1.367; 95% CI: 1.114, 1.676) than manual workers. A family history of dyslipidemia increased the likelihood of awareness (OR = 3.620; 95% CI: 2.816, 4.653) and treatment (OR = 3.298; 95% CI: 2.488, 4.371) of dyslipidemia. Alcohol drinking and physical activity were associated with a lower level of awareness and treatment.

Cigarette smokers (OR = 0.501; 95% CI: 0.349, 0.719) and those with BMI ≥ 24 kg/m^2^ (OR = 0.480; 95% CI: 0.326, 0.706) who received treatment were also associated with poor dyslipidemia control.

**Conclusion:**

Our study highlights low levels of awareness, poor treatment and control of dyslipidemia among adults aged 18 ~ 79 in the Jilin province. Promotion of healthy lifestyles and establishment of a comprehensive strategy of screening, treatment and control of dyslipidemia is needed to reduce or prevent the risk of cardiovascular disease in the Jilin province.

## Introduction

In China, the prevalence of dyslipidemia among adults increases yearly and dyslipidemia is now an important risk factor for cardiovascular diseases in the Chinese population [1,2]. The results from the Chinese National and Health Survey in 2002 showed that the prevalence of dyslipidemia in the Chinese population was 18.6% [3]. The study of Wang et al. [4] reported that the prevalence of dyslipidemia in the Beijing adult population was 56.1% in 2006. Luo et al. [5] reported that prevalence of dyslipidemia was 52.72% among adults in northwestern China in 2010. CVD is characterized by high morbidity of stroke and low morbidity of coronary heart disease, but morbidity and mortality of coronary heart diseases have gradually increased in the last 20 years in China [6]. The report of the third recalled sample survey on the causes of death in China showed that in the last 15 years, the proportion of deaths from chronic diseases among residents increased from 76.5% to 82.5% among residents, and cardiovascular death was the first cause [7]. It is apparent that China will continue to experience significant increase in the prevalence of cardiovascular-related morbidity and mortality, which has the potential to create an enormous burden on the health care system in the future [8].

Moreover, the asymptomatic characteristics of dyslipidemia contribute to the increased incidence of CVD and sudden death, thus uncontrolled dyslipidemia is a serious risk factor for cardiovascular events such as coronary heart disease, cerebral ischemic attack, cerebral infarction and peripheral vascular disease [9,10]. Large clinical trials have demonstrated that the treatment of dyslipidemia is effective in both primary and secondary prevention of CVD [11,12]. Treating dyslipidemia can reduce the risk of heart diseases by approximately 30% over a 5-year period [12]. Surveys in China have showed that the awareness, treatment and control of dyslipidemia is poor [1,8,13], and only limited studies on the influencing factors associated with the awareness, treatment and control of dyslipidemia in China have been carried out.

The aims of this study were: (1) to estimate the awareness, treatment, and control of dyslipidemia in the adult population in the Jilin province; and (2) to determine the influence factors associated with the awareness, treatment and control of dyslipidemia in the adult population in the Jilin province.

## Materials and methods

### Ethical statement

The study was approved by the Institutional Review Board of the School of Public Health, Jilin University, and all subjects participated in the study after making signed informed consents.

### Study population

Data were obtained from the survey of chronic disease and risk factors among adults in the Jilin province of China in 2012. The investigation was a cross-sectional study that aimed to assess chronic disease and associated risk factors among different populations in different regions of the Jilin province in China. The survey used a multistage cluster random sampling method to select a representative sample of permanent residents aged 18 to 79 years in nine different cities in the Jilin province. The detailed sampling process has been published elsewhere [14]. A total of 17,729 participants completed the survey and examination after excluding some participants due to incomplete blood lipid information. Among the 17,729 respondents, a total of 7,138 subjects had dyslipidemia and were included in the study.

### Data collection

#### Questionnaire interview

The study used a personal health survey questionnaire established by the School of Public Health, Jilin University. A direct face-to-face questionnaire interview was carried out by uniformly trained investigators.

The questionnaire provided the demographic information (such as region, age, gender, level of education, occupation), health behaviors (such as smoking, drinking, diet, physical activity), history of dyslipidemia in the past one year, and the current treatment of dyslipidemia.

#### Anthropometric

After the questionnaire interview, participants underwent anthropometric checks including height and weight. Weight was measured early in the morning before respondents had eaten, and each physical measurement was completed by two people together.

#### Laboratory assay

Fasting blood samples of each respondent were drawn by venipuncture to measure serum Total Cholesterol (TC), High Density Lipoprotein Cholesterol (HDL-C), Low Density Lipoprotein Cholesterol (LDL-C) and Triglycerides (TG). The tools used for all blood samples collection were from the same source. After collection, the samples were placed in a cold chain system before being collectively transported to a central laboratory. Blood samples were analyzed using MODULE P800 automated biochemistry analyzer (ROCHE, USA).

### Definitions

Dyslipidemia was defined according to the Chinese guidelines on the prevention and treatment of dyslipidemia in adults (2007) [6]: TC ≥ 6.22 mmol/L (240 mg/dL) as high; LDL-C ≥ 4.14 mmol/L (160 mg/dL) as high; HDL-C < 1.04 mmol/L (40 mg/dL) as low; and TG ≥ 2.26 mmol/L (200 mg/dL) as high. In this study, high TC, and/or high LDL-C, and/or low HDL-C and/or high TG, and/or having a history of dyslipidemia disease in the past one year, and/or currently receiving treatment with lipid-lowering medications was regarded as dyslipidemia.

Awareness of dyslipidemia was defined as a self-reported diagnosis of dyslipidemia by a healthcare professional or treatment with medications within the population defined as having dyslipidemia. Treatment of dyslipidemia was defined as using medications to treat dyslipidemia among participants with dyslipidemia. Dyslipidemia was considered to be controlled among the population defined as having dyslipidemia and being treated with medication if TC < 6.22 mmol/L, LDL-C < 4.14 mmol/L, HDL-C ≥ 1.04 mmol/L, and TG < 2.26 mmol/L.

Body mass index (BMI) was calculated as weight (kg) divided by height (m^2^). Using BMI 24 kg/m^2^ as cut off point, participants were broadly categorized into two main groups: overweight or obesity being defined as BMI ≥ 24 kg/m^2^, and underweight or normal being defined as BMI < 24 kg/m^2^. Smoking was defined as having smoked at least one cigarette per day in the past 30 days, or past smokers even if they had exhibited complete abstinence from cigarette use for at least one month [8,15]. Drinking was defined as drinking any kind of purchased or homemade alcohol-containing beverages on average more than once a week. Physical activity was defined as walking, running, going to the gym or other conscious exercise not less than once a week. Participants with a family history of dyslipidemia and those who like to eat animal-based foods and salted foods were ascertained by questionnaire.

### Statistical analysis

EpiData3.1 was used to establish a database from which and the data was then exported to SPSS 16.0 software for further statistical analyses. *Chi-square (χ*^*2*^*)* test was used to compare the rates of dyslipidemia awareness, treatment and control between different characteristics of the participants. Multiple logistic regression analyses were performed separately for each group to explore the associations between participants’ characteristics and dyslipidemia awareness, treatment and control. All statistical tests were two-tailed and *P*-values ≤ 0.05 considered statistically significant.

## Results

### Characteristics of the study sample

Table 1 presents the characteristics of the dyslipidemia subjects. Out of the 7319 study subjects, 50.1% were from the urban region, 51.1% were men, 48.9% were women, 30.4% were aged between 45 and 54, the mean age was 50.50 ± 12.30 years, 69.0% attained a junior high school or higher education, 88.0% were married, and 52.4% were manual workers. In terms of BMI distribution, 67.9% were BMI ≥ 24 kg/m^2^. Only 4.9% had a family history of dyslipidemia. The rate of smoking was 44.2%, and drinking was 33.7%, with 9.2% admitted eating more of animal-based foods, 40.4% more of salted foods, and 42.3% of the study subjects reported having regular exercises.

**Table 1:** Characteristics of the study sample (n = 7319) 2012, Jilin province, China

**Variables**	**N**	**%**
Regions		
Urban	3653	50.1
Rural	3666	49.9
Gender		
Male	3740	51.1
Female	3579	48.9
Age group (years)		
18~	821	11.2
35~	1388	19.0
45~	2225	30.4
55~	1987	27.1
65 ~ 79	898	12.3
Education		
Primary school or low	2269	31.0
Junior high school	2090	28.6
Senior high school	1956	26.7
University	1004	13.7
Marital status		
Married	6444	88.0
Single/Divorced/Widowed	875	12.0
Occupation		
Manual workers	3832	52.4
Mental workers	1297	17.7
Retired	2190	29.9
BMI ≥ 24 kg/m^2^		
No	2346	32.1
Yes	4973	67.9
Family history of dyslipidemia		
No	6959	95.1
Yes	360	4.9
Smoking		
No	4087	55.8
Yes	3232	44.2
Drinking		
No	4853	66.3
Yes	2446	33.7
Like to eat animal-based foods		
No	6647	90.8
Yes	672	9.2
Like to eat salted foods		
No	4363	59.6
Yes	2956	40.4
Physical activity		
No	4222	57.7
Yes	3097	42.3

### Awareness, treatment and control of dyslipidemia

Table 2 shows the prevalence of dyslipidemia awareness, treatment and control. Among participants with dyslipidemia, 852 (11.6%) were aware of the diagnosis of their condition, and the overall rate of dyslipidemia treatment was 8.4%. Among the 615 dyslipidemia patients receiving medical treatment for dyslipidemia, 214 (34.8%) were under control.

**Table 2:** Awareness, treatment and control of dyslipidemia by characteristics, 2012, Jilin province, China

**Variables**	**Awareness**^**a**^		**Treatment**^**a**^		**Control**^**b**^	
	**N(%)**	***P***	**N(%)**	***P***	**N(%)**	***P***
Total	852(11.6)		615(8.4)		214(34.8)	
Residential areas		0.083		0.863		0.075
Urban	449(12.3)		309(8.5)		97(31.4)	
Rural	403(11.0)		306(8.3)		117(38.2)	
Gender		0.018		0.014		0.058
Male	403(10.8)		285(7.6)		88(30.9)	
Female	449(12.5)		330(9.2)		126(38.2)	
Age group (years)		<0.001		<0.001		0.886
18~	24(2.9)		15(1.8)		4(26.7)	
35~	130(9.4)		87(6.3)		33(37.9)	
45~	283(12.7)		201(9.0)		72(35.8)	
55~	285(14.3)		211(10.6)		70(33.2)	
65 ~ 79	130(14.5)		101(11.2)		35(34.7)	
Education		0.006		0.146		0.075
Primary school or lower	225(9.9)		168(7.4)		63(37.5)	
Junior high school	249(11.9)		192(9.2)		71(37.0)	
Senior high school	238(12.2)		163(8.3)		59(36.2)	
University	140(13.9)		92(9.2)		21(22.8)	
Marital status		0.058		0.328		0.993
Married	767(11.9)		549(8.5)		191(34.8)	
Single	85(9.7)		66(7.5)		23(34.8)	
Occupation		<0.001		<0.001		0.529
Manual workers	351(9.2)		253(6.6)		92(36.4)	
Mental workers	169(13.0)		109(8.4)		33(30.3)	
Retired	332(15.2)		253(11.6)		89(35.2)	
BMI ≥ 24 kg/m^2^		<0.001		<0.001		<0.001
No	206(8.8)		144(6.1)		69(47.9)	
Yes	646(13.0)		471(9.5)		145(30.8)	
Family history of dyslipidemia		<0.001		<0.001		0.948
No	745(10.7)		541(7.8)		188(34.8)	
Yes	107(29.7)		74(20.6)		26(35.1)	
Smokers		<0.001		0.004		<0.001
No	524(12.8)		377(9.2)		153(40.6)	
Yes	328(10.1)		238(7.4)		61(25.6)	
Drinking		<0.001		<0.001		0.082
No	616(12.7)		454(9.4)		167(36.8)	
Yes	236(9.6)		161(6.5)		47(29.2)	
Like to eat animal-based foods		0.055		0.094		0.912
No	789(11.9)		570(8.6)		198(34.7)	
Yes	63(9.4)		45(6.7)		16(35.6)	
Like to eat salted foods		0.184		0.035		0.061
No	490(11.2)		342(7.8)		130(38.0)	
Yes	362(12.2)		273(9.2)		84(30.8)	
Physical activity		<0.001		<0.001		0.689
No	587(13.9)		423(10.0)		145(34.3)	
Yes	265(8.6)		192(6.2)		69(35.9)	

^a^among all dyslipidemia subjects.

^b^among those who received treatment.

#### Awareness of dyslipidemia

The awareness of dyslipidemia increased with age (*P* < 0.001), with the last age group 65 ~ 79 having the highest level of awareness, 130(14.5%). Awareness of dyslipidemia also differed significantly by gender with females being more aware of their dyslipidemia than males (*P* = 0.018); education with participants who attained university level of education being more aware of their condition compared to lower levels of education (*P* = 0.006); There were significant differences between the occupations in awareness of dyslipidemia (*P* < 0.001), with retired having the highest level of awareness, 332(15.2%). BMI with subjects having BMI ≥ 24 kg/m^2^ being more aware than those with BMI < 24 kg/m^2^ (*P* < 0.001); and participants with family history of dyslipidemia had higher levels of awareness of dyslipidemia compared to those without any history of the condition (*P* < 0.001). Smoking, drinking and physical activity were associated with higher levels of dyslipidemia awareness (all *P* < 0.01). The awareness of dyslipidemia was not significantly associated with residential area (*P* = 0.083), marital status (*P* = 0.058), or whether the participants like more of animal-based foods (*P* = 0.055) or salted foods (*P* = 0.184).

#### Treatment of dyslipidemia

The treatment rate increased with age (*P* < 0.001), with the last age group 65 ~ 79 having the highest rate of treatment, 101(11.2%); and differed significantly by gender (*P* = 0.014) and occupation (*P* < 0.001) with females and retired having higher treatment rates compared to males and manual workers respectively. The level of dyslipidemia treatment was also significantly associated with BMI and a family history of dyslipidemia (all *P* < 0.001) with subjects with BMI ≥ 24 kg/m^2^ and those with a family history of dyslipidemia having higher treatment rates as against those with BMI < 24 kg/m^2^ or without a family history of dyslipidemia respectively. Smoking (*P* = 0.004), drinking (*P* < 0.001), liking to eat salted foods (*P* = 0.035) and physical activity (*P* < 0.001) were all associated with higher levels of dyslipidemia treatment. In this present study, treatment of dyslipidemia was not found to be significantly associated with residential area (*P* = 0.863), level of education *(P* = 0.146), marital status (*P* = 0.328), or liking to eat animal-based foods (*P* = 0.094) or salted foods (*P* = 0.035).

#### Control of dyslipidemia

Among dyslipidemia patients receiving medical treatment for dyslipidemia, participants with BMI < 24 kg/m^2^ were more likely to have their dyslipidemia controlled than those with BMI ≥ 24 kg/m^2^ (*P* < 0.001). Smoking was associated with a lower level of dyslipidemia control than non-smoking (*P* < 0.001).

### Factors associated with awareness, treatment and control of dyslipidemia

In a multivariate binary logistic regression analysis, awareness, treatment, and control of dyslipidemia were used as dependent variables; and the parameters which were significantly associated with awareness, treatment, and control of dyslipidemia in the *χ*^2^ test (*P* < 0.05 in Table 2), were used as independent variables respectively.

As shown in Table 3, the prevalence of the awareness of dyslipidemia was significantly associated with increasing age, and there was a significant association between education and the awareness of dyslipidemia. Retired participants more likely to aware of their dyslipidemia condition than manual workers (OR = 1.255; 95% CI: 1.046, 1.506). Subjects with BMI ≥ 24 kg/m^2^ tended to be more aware of their dyslipidemia condition than those with BMI < 24 kg/m^2^ (OR = 1.547; 95% CI: 1.307,1.832). In addition, having a family history of dyslipidemia increased the tendency of awareness of dyslipidemia (OR = 3.620; 95% CI: 2.816, 4.653). Drinkers (OR = 0.780; 95% CI: 0.658, 0.925) or those who regularly do physical exercises (OR = 0.714; 95% CI: 0.606, 0.842) were unlikely to be aware of their dyslipidemia condition.

**Table 3:** Multivariate logistic regression analyses on influence factors for awareness of dyslipidemia, 2012, Jilin province, China

**Variables**	**Awareness**^**a**^		
	**OR**	**95% CI**	***P***
Age group (years)			
18-	1.000		
35-	3.865	2.464,6.062	<0.001
45-	5.481	3.554,8.453	<0.001
55-	6.491	4.170,10.105	<0.001
65-79	6.420	3.989,10.332	<0.001
Education			
Primary school or lower	1.000		
Junior high school	1.425	1.165,1.743	0.001
Senior high school	1.361	1.095,1.691	0.005
University	1.835	1.366,2.466	<0.001
Occupation			
Manual workers	1.000		
Mental workers	1.083	0.848,1.384	0.521
Retired	1.255	1.046,1.506	0.014
BMI ≥ 24 kg/m^2^			
No	1.000		
Yes	1.547	1.307,1.832	<0.001
Family history of dyslipidemia			
No	1.000		
Yes	3.620	2.816,4.653	<0.001
Drinking			
No	1.000		
Yes	0.780	0.658,0.925	0.004
Physical activity			
No	1.000		
Yes	0.714	0.606,0.842	<0.001

Method: Forward: LR; OR = odds ratio; CI = confidence interval.

^a^among all dyslipidemia subjects.

Table 4 illustrates that increasing age was significantly associated with the treatment of dyslipidemia, with retired (OR = 1.367; 95% CI: 1.114, 1.676) being more likely to be treated than manual workers. The higher the level of BMI the more likely that the respondent will receive treatment for dyslipidemia (OR = 1.575; 95% CI: 1.294, 1.918). Subjects with a family history of dyslipidemia were more likely than those without family history to report drug treatment among participants with dyslipidemia (OR = 3.298; 95% CI: 2.488, 4.371).

**Table 4:** Multivariate logistic regression analyses on influence factors for treatment of dyslipidemia, 2012, Jilin province, China

**Variables**	**Treatment**^**a**^		
	**OR**	**95% CI**	***P***
Age group (years)			
18-	1.000		
35-	3.816	2.184,6.669	<0.001
45-	5.338	3.126,9.112	<0.001
55-	6.043	3.519,10.377	<0.001
65-79	6.064	3.425,10.727	<0.001
Occupation			
Manual workers	1.000		
Mental workers	1.183	0.923,1.515	0.184
Retired	1.367	1.114,1.676	0.003
BMI ≥ 24 kg/m^2^			
No	1.000		
Yes	1.575	1.294,1.918	<0.001
Family history of dyslipidemia			
No	1.000		
Yes	3.298	2.488,4.371	<0.001
Drinking			
No	1.000		
Yes	0.756	0.620,0.923	0.006
Like to eat salted foods			
No	1.000		
Yes	1.319	1.110,1.567	0.002
Physical activity			
No	1.000		
Yes	0.683	0.567,0.824	<0.001

Method: Forward: LR; OR = odds ratio; CI = confidence interval.

^a^among all dyslipidemia subjects.

As shown in Table 5, among the study subjects with dyslipidemia who were treated with drugs, BMI ≥ 24 kg/m^2^ was associated with poor control of dyslipidemia (OR = 0.480; 95% CI: 0.326, 0.706). Smokers were less likely to control their serum lipids at normal levels (OR = 0.501; 95% CI: 0.349, 0.719).

**Table 5:** Multivariate logistic regression analyses on influence factors for control of dyslipidemia, 2012, Jilin province, China

**Variables**	**Control**^**b**^		
	**OR**	**95% CI**	***P***
BMI ≥ 24 kg/m^2^			
No	1.000		
Yes	0.480	0.326,0.706	<0.001
Smoking			
No	1.000		
Yes	0.501	0.349,0.719	<0.001

Note: Method: Forward: LR; OR = odds ratio; CI = confidence interval.

^b^among those who receiving treatment.

## Discussions

With economic growth and associated changes in lifestyle and diet, the level of serum cholesterol in the Chinese population has greatly increased [4,16]. Dyslipidemia is a major pathogenic factor of atherosclerosis, and one of the independent risk factors for cardiovascular disease such as coronary heart disease and stroke [1,5]. Increasing the awareness and management of patients with dyslipidemia has a positive impact on cardiovascular disease prevention [11]. Despite this, numerous studies have revealed poor awareness and unsatisfactory treatment and control in many European countries [17], and extremely low rates of dyslipidemia in the Chinese population [3,6,8,13]. It has been reported that the awareness, treatment, and control rates of dyslipidemia were comparatively low among Chinese adults aged 18 and above, at 10.93%, 6.84% and 35.3% respectively in 2010 [1]. We found similar results in this present study with rates of awareness and treatment among participants being 11.6% and 8.4% respectively. However, the rates in our study were significantly less than the 22.2% awareness of diagnosis and 46.1% receiving treatment among adults in Beijing, China in 2012 [8]. Out of the 615 dyslipidemia individuals in our study that were receiving treatment, 34.8% were under control. This is slightly lower than the 37.8% serum lipids control finding of Cai et al. [8] among those with dyslipidemia receiving treatment. This indicates that dyslipidemia is an important health risk factors in the Jilin province. However, only limited studies on the influencing factors associated with awareness, treatment and control of dyslipidemia had been carried out in China. Hence our study aimed to examine the awareness, treatment, and control of dyslipidemia, and their influence factors in the Jilin province.

Because dyslipidemia is almost asymptomatic and its detection requires blood analysis which in most cases requested by a physician, ordinary residents are hardly aware of and being treated for dyslipidemia disease [17]. The results of our study revealed that the awareness and treatment of dyslipidemia among our study population increased concomitantly with age. This is in line with other studies such as the rate of awareness among residents in Beijing being less than 15% among adults aged 45 and above [18], and the awareness rate also increasing with age among residents in Laiwu city, China [19]. This means that as people advance in age, they become more concerned about their health, particularly being concerned about cardiovascular diseases, than younger individuals who are less likely to attach great importance to disease consciousness.

Several recent studies showed that education is positively associated with the awareness of dyslipidemia [10,18,20,21]. The more an individual attains higher levels of education, the more likely awareness about health conditions, including dyslipidemia, increases. Regarding the determinants of awareness in our study, an increasing education level was associated with a higher level of dyslipidemia awareness.

Our study also showed that compared with manual workers, retired participants were more likely to be aware of their dyslipidemia condition and seek treatment. This may be related to retirees, students having more time to focus on their health, and tend to seek early management of any adverse health condition. However, manual workers always appear body fatigue after a whole day’s work, so they easily ignore the condition body health.

Obesity is an independent risk factor of dyslipidemia [17,22]. Findings from the ORISCAV-LUX study revealed that obese subjects are more conscious of cardiovascular health risks than slim individuals. This is helpful in increase their awareness of the underlying silent metabolic pathologies associated with excess body weight [17]. Our findings are consistent with other studies that overweight or obesity (BMI ≥ 24 kg/m^2^) is associated with higher levels of dyslipidemia awareness and treatment [19,20,23]. Individuals with BMI ≥ 24 kg/m^2^ tend to control their BMI at 24 kg/m^2^ or less, which helps in preventing 50-60% risk of hypertriglyceridemia in this population [24]. However, among individuals receiving treatment, individuals with BMI ≥ 24 kg/m^2^ are less likely to have controlled dyslipidemia. This finding is similar to the results of Long’s study in 2007 [22]. The plausible explanation is that weight control is a lengthy process, and it is more difficult to control blood lipids in overweight or obese people.

People with a family history of dyslipidemia have a higher risk of developing dyslipidemia [1]. In this study, we found that family history of dyslipidemia was a strong predictor of dyslipidemia awareness and treatment. Unsurprisingly, having a family member with dyslipidemia would increase the consciousness and alertness of the whole family with regards to dyslipidemia, and physicians tend to pay more attention to patients with a family history of dyslipidemia, as they tend to have increased risk of cardiovascular diseases.

Consistent with other findings [19], our study revealed physical activity is associated with lower levels of awareness and treatment of dyslipidemia. This may be explained by the fact that people who exercise regularly believe they are less likely to become sick, and therefore are less likely to be conscious of dyslipidemia.

Lower levels of awareness and treatment were found among alcohol drinkers and also lower levels of dyslipidemia control among cigarette smokers in the present study. This may be explained that the dyslipidemia patients who are alcohol drinkers and cigarette smokers usually receive advice on drinking and smoking cessation given by physicians, or it may be associated with alcohol drinkers and smokers’ lower level of concern about their own health [18,25]. Several studies have found that lifestyle changes are effective in controlling serum blood lipids [8,19,26]. Thus it is important to emphasize the need for policies that improve healthy lifestyle.

### Study limitations

The findings should be interpreted with an understanding of the following potential limitations. The cross-sectional nature of our study design means that causal associations can only be made with caution. As in many surveys, our serum lipid levels and the definitions of dyslipidemia awareness, treatment, and control were based on measurements taken during a single visit. Potential sources of bias include recall bias of self-reported information. Treatment and control rates were based on only pharmacological treatment of dyslipidemia, however, it is possible to control serum lipid by non-pharmacological means, such as diet and/or exercise. The Chinese criteria were adopted to define dyslipidemia; therefore, we could not directly compare our results with those from other countries.

## Conclusions

In conclusion, our study highlighted the low rates of awareness, treatment and control of dyslipidemia among adults aged 18–79 in the Jilin province, China. Continuous efforts are needed to increase the awareness, treatment and control of dyslipidemia taking influence factors into account. Our results suggest that the relevant departments should regularly carry out health education programs on dyslipidemia. These departments should also provide guidelines that help promote healthy lifestyles such as increased physical activity, weight control, smoking cessation and cessation of alcohol drinking. Establishment of a comprehensive strategy of screening, treating and controlling of dyslipidemia is needed in the Jilin Province. These interventions will help to reduce the high and increasing burden of cardiovascular disease in the Jilin province, China.

## Abbreviations

CVD: Cardiovascular disease; TC: Total cholesterol; HDL-C: HDL cholesterol; LDL-C: LDL cholesterol; TG: Triglycerides; BMI: Body mass index.

## Competing interests

The authors declare that they have no competing interests.

## Authors’ contributions

YQY, YWL, CGK, BL, YCT, QZ, HH and CW designed the study and participated in acquisition of data; HH, CW and YL researched and evaluated the literature; HH undertook the statistical analysis and wrote the first draft of the manuscript. YWL, JSK, XFH and MH revised the manuscript critically for important intellectual content and languages. All authors have approved the final manuscript for publication.
